# Evaluation of the indirect impact of the 10-valent pneumococcal *Haemophilus influenzae* protein D conjugate vaccine in a cluster-randomised trial

**DOI:** 10.1371/journal.pone.0261750

**Published:** 2022-01-05

**Authors:** Hanna Rinta-Kokko, Arto A. Palmu, Esa Ruokokoski, Heta Nieminen, Marta Moreira, Lode Schuerman, Dorota Borys, Jukka Jokinen

**Affiliations:** 1 Department of Public Health and Welfare, Finnish Institute for Health and Welfare, Helsinki, Finland; 2 Department of Public Health and Welfare, Finnish Institute for Health and Welfare, Tampere, Finland; 3 Department of Information Services, Finnish Institute for Health and Welfare, Helsinki, Finland; 4 GSK, Wavre, Belgium; Public Health England, UNITED KINGDOM

## Abstract

**Background:**

In the nation-wide double-blind cluster-randomised Finnish Invasive Pneumococcal disease trial (FinIP, ClinicalTrials.gov NCT00861380, NCT00839254), we assessed the indirect impact of the 10-valent pneumococcal *Haemophilus influenzae* protein D conjugate vaccine (PHiD-CV10) against five pneumococcal disease syndromes.

**Methods:**

Children 6 weeks to 18 months received PHiD-CV10 in 48 clusters or hepatitis B/A-vaccine as control in 24 clusters according to infant 3+1/2+1 or catch-up schedules in years 2009―2011. Outcome data were collected from national health registers and included laboratory-confirmed and clinically suspected invasive pneumococcal disease (IPD), hospital-diagnosed pneumonia, tympanostomy tube placements (TTP) and outpatient antimicrobial prescriptions. Incidence rates in the unvaccinated population in years 2010―2015 were compared between PHiD-CV10 and control clusters in age groups <5 and ≥5 years (5―7 years for TTP and outpatient antimicrobial prescriptions), and in infants <3 months. PHiD-CV10 was introduced into the Finnish National Vaccination Programme (PCV-NVP) for 3-month-old infants without catch-up in 9/2010.

**Results:**

From 2/2009 to 10/2010, 45398 children were enrolled. Vaccination coverage varied from 29 to 61% in PHiD-CV10 clusters. We detected no clear differences in the incidence rates between the unvaccinated cohorts of the treatment arms, except in single years. For example, the rates of vaccine-type IPD, non-laboratory-confirmed IPD and empyema were lower in PHiD-CV10 clusters compared to control clusters in 2012, 2015 and 2011, respectively, in the age-group ≥5 years.

**Conclusions:**

This is the first report from a clinical trial evaluating the indirect impact of a PCV against clinical outcomes in an unvaccinated population. We did not observe consistent indirect effects in the PHiD-CV10 clusters compared to the control clusters. We consider that the sub-optimal trial vaccination coverage did not allow the development of detectable indirect effects and that the supervening PCV-NVP significantly diminished the differences in PHiD-CV10 vaccination coverage between the treatment arms.

## Introduction

The indirect effects of a vaccination programme are the protective or detrimental effects that are mediated by the intervention-induced changes in transmission [1]. Herd immunity develops due to reduced transmission of the causative pathogen in the population, either by lowering the number of sick subjects transmitting the disease or by reducing the number of subjects carrying the causative pathogen and the subsequent opportunities for further transmission and progression to disease [2]. While the immunological direct effect in the vaccinated population develops within a couple of weeks, the indirect effects develop more slowly after dynamic transmission cascades.

*Streptococcus pneumoniae* has about 100 circulating serotypes [3] and the currently available pneumococcal conjugate vaccines (PCVs) contain capsular polysaccharides of 10 to 13 serotypes [4]. The protective effects usually apply only to the serotypes included in the vaccines, while non-vaccine serotypes may replace the vaccine types in nasopharyngeal carriage and at least partly in disease [5]. Thus, PCV indirect effects are composed of both herd immunity and serotype replacement that affect both the vaccinated and unvaccinated population [2, 6].

Given the documented efficacy of PCVs on vaccine-type nasopharyngeal carriage [7], it was expected to see some degree of indirect effects also on pneumococcal disease outcomes [6]. Some indirect effects on the unvaccinated populations were detected in observational studies after the large-scale introduction of the 7-valent PCV (PCV7) in infant programmes in 2000s [8–10], and later after the introduction of the higher valent PCVs [11–18]. As observational studies with historical reference incidence are prone to many sources of bias, such as secular trends, indirect effects may be difficult to assess. The cluster-randomised design mimics vaccination programmes and enables the evaluation of the indirect impact in unvaccinated populations when all children within a cluster are vaccinated with the study vaccine and compared to unvaccinated populations in control clusters in parallel [19, 20]. However, to our knowledge there have been no clinical trial data to demonstrate the development and extent of the indirect effects of the PCVs on pneumococcal clinical outcomes.

We planned and conducted the cluster-randomised nation-wide double-blind Finnish Invasive Pneumococcal disease (FinIP) trial enabling the evaluation of the indirect effects in addition to the total effects of the 10-valent pneumococcal *Haemophilus influenzae* protein D conjugate vaccine (PHiD-CV10, *Synflorix*, GSK) [21]. Here, we present the evaluation of the indirect impact in the unvaccinated population from 2010 to 2015 following vaccination of 6-week-old to 18-month-old children in the PHiD-CV10 clusters compared to the control clusters. Although we observed some signals of indirect effects, we did not observe consistent indirect impact in this clinical trial setting.

## Materials and methods

### Study design

**Fig 1:**
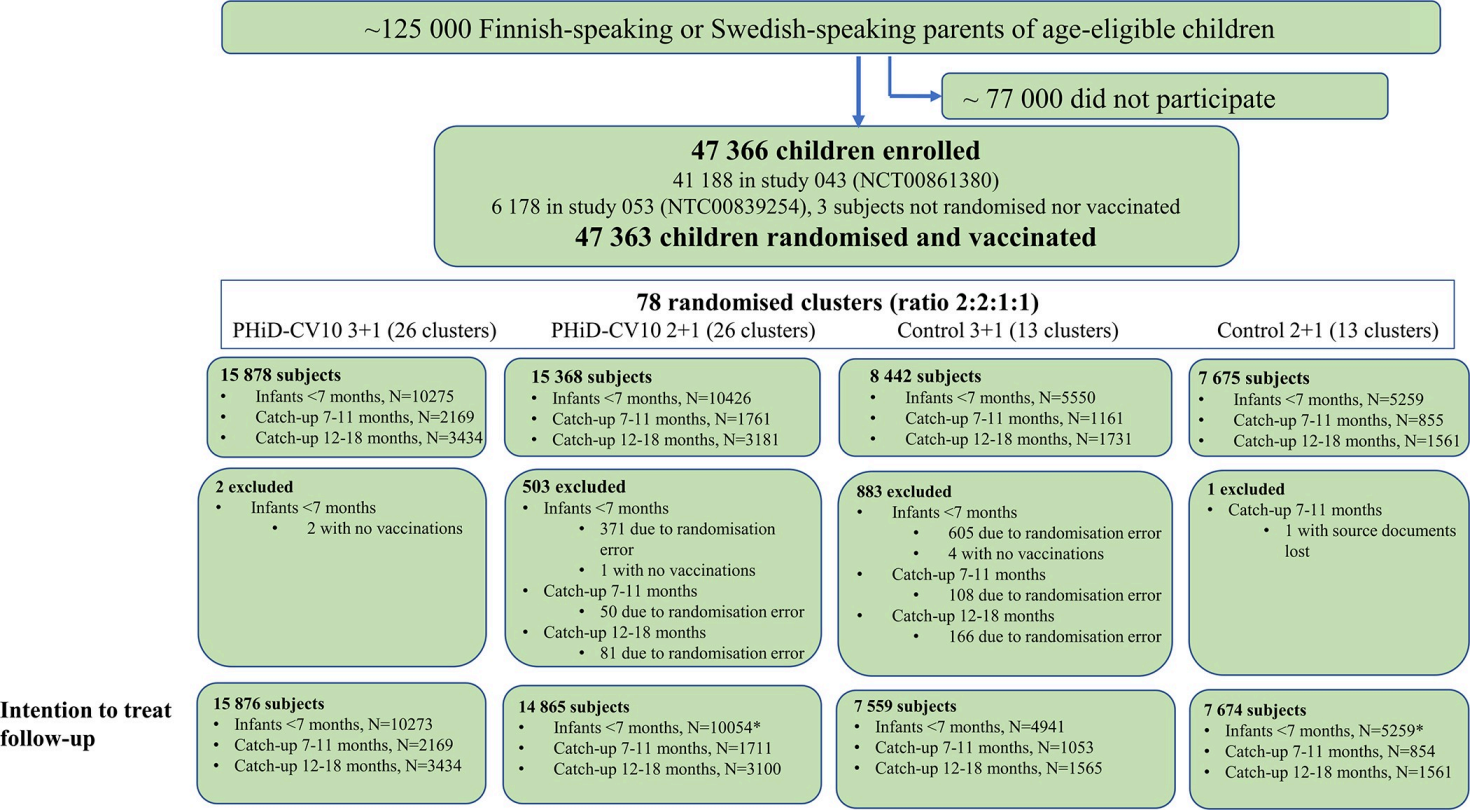
Trial profile of 78 clusters. The 3+1 and 2+1 clusters differed only for the infant schedules. Catch-up schedules were identical for the 3+1 and 2+1 clusters and were always combined for the analyses. For the indirect effect analysis, 72 clusters were included. * = Includes one subject withdrawn from the register follow-up during the blinded follow-up period. The figure has been presented previously [23].

The FinIP trial was a phase III/IV cluster-randomised double-blind trial (ClinicalTrials.gov NCT00861380). The study design has been published previously [21]. Briefly, children 6 weeks to 18 months of age received PHiD-CV10 in 52 clusters or control hepatitis vaccines in 26 clusters. Infants 6 weeks to 6 months of age at the first vaccination received either a 3+1 or a 2+1 vaccination schedule, while children 7 to 11 and 12 to 18 months of age received 2+1 and 2-dose catch-up schedules, respectively. The control clusters used the same vaccination schedules as above with hepatitis B vaccine (*Engerix-B*, GSK) as control for infants 6 weeks to 11 months of age and hepatitis A vaccine (*Havrix 720 Junior*, GSK) as control for children 12 to 18 months of age. From February 18, 2009, to October 5, 2010, 47366 children were enrolled in 651 local well-baby clinics, including 15 Tampere University Vaccine Research Centre (TAUVRC) clinics, and vaccinated during routine visits. TAUVRC contributed to study enrolment in a parallel trial, nested within the FinIP trial design (ClinicalTrials.gov NCT00839254) [22]. The trial enrolment ended, as planned, when PHiD-CV10 was introduced into the Finnish National Vaccination Programme (PCV-NVP) for 3-month-old infants in September 2010 in a 2+1 schedule without catch-up vaccinations of older children. Last trial vaccine doses were administered in September 2011. Written informed consent was obtained from a parent or legal guardian. Participants could be enrolled provided they had not received and were not anticipated to receive any of the study vaccines, nor had any study-vaccine-specific or general contraindications to immunisations. The trial profile is presented in Fig 1. The demographic details of the study participants have been described previously [21].

For the indirect impact assessment, we included 72 study clusters based on geographical areas of the participating health centers (S1 Fig). TAUVRC contributed to the study enrolment in 44 of these clusters in the biggest cities [22]. The six additional clusters where the enrolment was conducted only through TAUVRC clinics were not included in the evaluation of the indirect impact due to low enrolment proportions. The 72 clusters were randomised (2:2:1:1) into PHiD-CV10 3+1, PHiD-CV10 2+1, control 3+1 and control 2+1 groups.

The population of the 72 clusters that was not vaccinated in the FinIP trial was included in the evaluation of the indirect impact. The use of PCVs (PCV7, PHiD-CV10 and 13-valent PCV [PCV13] licensed in 2001, 2009, and 2009 respectively in the European Union) was negligible (<1%) among infants and children before the FinIP trial. With regard to adults, the vaccination coverage of the 23-valent polysaccharide vaccine (PPV23) has been low over years: data from the Health 2011 [24] obtained by personal interviews indicate that <2% of the elderly population 65 years or older have ever received PPV23 in Finland. The cumulative vaccination coverage of PCV13, which was licensed for use in adults in November 2011, was 8.9% in 2015 in the age group ≥65 years [25]. PCV13-vaccinated individuals were not excluded from the analysis, because they were assumed to be randomly allocated to PHiD-CV10 and control clusters.

### Outcomes

The outcomes and case definitions are summarised in Table 1 and have been presented in detail previously [21, 23, 26–28]. All outcome data were collected from established national health registers in routine use (Table 1). The Personal Identity Code was used to identify the cases for the current analysis and to exclude the cases in vaccinated FinIP participants. For each disease syndrome, a new episode was considered to start if a specific number of days had elapsed from the beginning of the previous one (see Table 1).

**Table 1:** Outcome data collected for the study.

Pneumococcal disease syndrome	Register source	Case definitions	Interval between episodes
IPD	National Infectious Diseases Register, THL	Laboratory-confirmed IPD: *Streptococcus pneumoniae* confirmed by culture and/or DNA/RNA detection from a normally sterile site.	90-day
		These cases were divided into mutually exclusive groups:	
		Vaccine-type IPD: IPD due to the 10 PHiD-CV10 serotypes.	
		Vaccine-related type IPD: IPD due to same serogroup as vaccine types.	
		Non-vaccine-related type IPD: IPD due to remaining serotypes.	
Clinically suspected IPD without laboratory confirmation	Care Register for Health Care, THL, and National Infectious Diseases Register, THL	Non-laboratory-confirmed IPD: a hospital physician’s diagnosis compatible with IPD (ICD-10 codes A40.3, B95.3, G00.1 or M00.1), but not confirmed by laboratory detection of *Streptococcus pneumoniae*.	90-day
		Non-laboratory-confirmed IPD or unspecified sepsis: as above and including ICD-10 codes for unspecified sepsis (A40.9, A41.9, A49.9, G00, G00.9, I30.1, M00, M00.9, or B95.5).	
Pneumonia	Care Register for Health Care, THL	Hospital-diagnosed pneumonia (HDP): a hospital physician’s diagnosis compatible with pneumonia (ICD-10 codes J10.0, J11.0, J12 to J18, J85.1 or J86).	90-day
		Hospital-treated primary pneumonia (HTPP): as above with the primary discharge diagnosis compatible with pneumonia after in-patient hospitalisation.	
		Empyema: ICD-10 code J86 with in-patient hospitalisation.	
Tympanostomy tube placement (TTP)*	Care Register for Heath Care, THL, and The Social Insurance Institution’s Benefits Register, KELA	NOMESCO code for TTP (DCA20).	1-day
Antimicrobial treatment*	The Social Insurance Institution’s Benefits Register, KELA	A purchase of an antimicrobial recommended by the Finnish guideline for treatment of acute otitis media including • amoxicillin (ATC-code J01CA04) • amoxicillin with enzyme inhibitor clavulanic acid (J01CR02) • phenoxymethylpenicillin (J01CE02) • cefuroxime (J01DC02) • cefaclor (J01DC04) • sulfadiazine and trimethoprim (J01EE02) • clarithromycin (J01FA09) • azithromycin (J01FA10)	1-day

*Surrogates for otitis media

Abbreviations: ATC, anatomical therapeutic chemical classification system; ICD-10, 10^th^ revision of the International Classification of Diseases; IPD, invasive pneumococcal disease; KELA, The Social Insurance Institution of Finland; NOMESCO, Nordic Medico-Statistical Committee; THL, Finnish Institute for Health and Welfare; TTP, tympanostomy tube placement.

### Study cohorts

The population of Finland was 5.3 million in 2010 out of whom 4.0 million (76%) lived in the 72 study cluster areas. For the evaluation of the indirect impact, the population of the 72 clusters that was not vaccinated in the FinIP trial was included in the analysis. For tympanostomy tube placements (TTPs) and outpatient antimicrobial prescriptions, children ≤7 years of age were included in the analysis.

Children vaccinated within the FinIP trial (including both PHiD-CV10 and control vaccine recipients) were excluded from the analyses starting from the date of the first vaccination. However, children eligible for PHiD-CV10 in the PCV-NVP (born June 1, 2010 or later) were not excluded in order to keep the age distributions similar in successive calendar years and because PHiD-CV10 coverage (first dose range 93.5―95.6% in birth cohorts of 2012―2015 [25], S2 Fig) was considered to be similar between the randomised study clusters.

**Fig 2:**
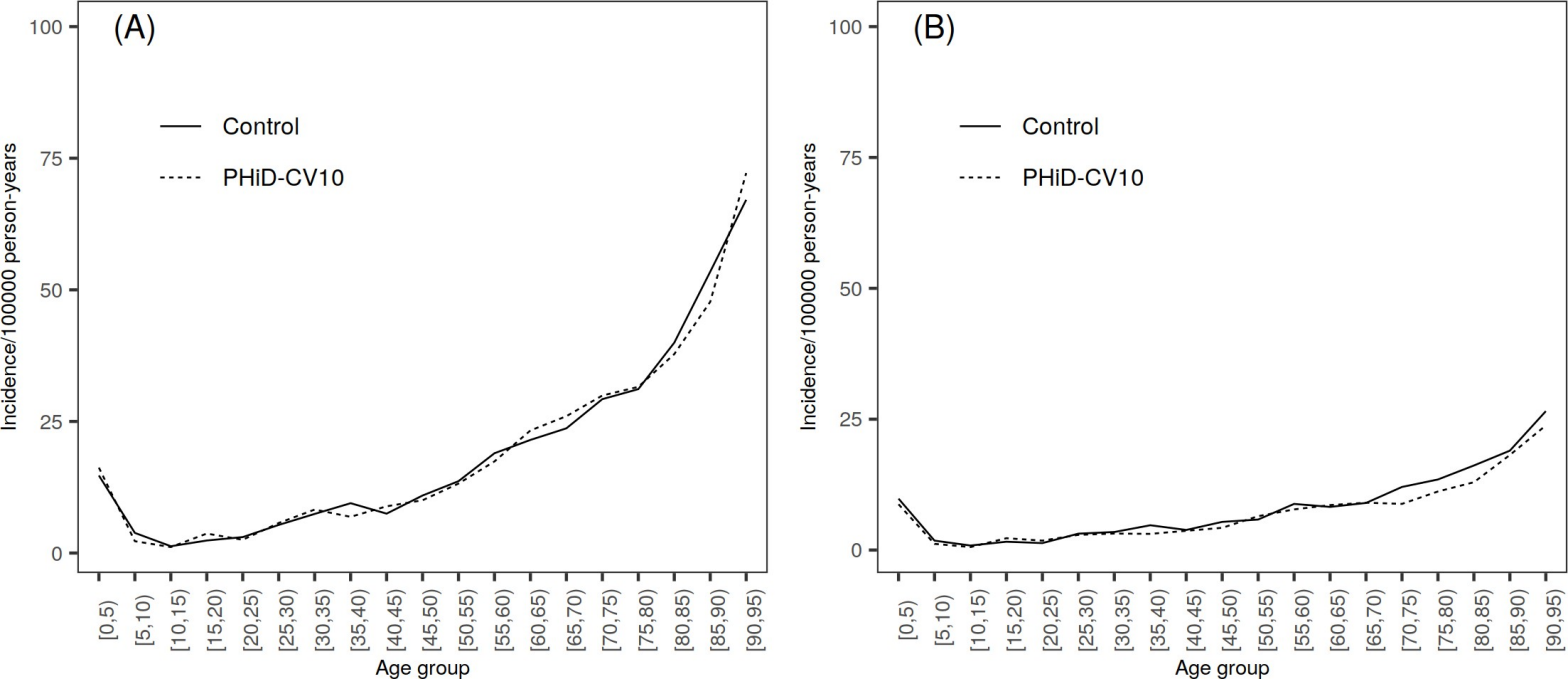
Incidence of IPD by age group (in years) in PHiD-CV10 and control clusters, average over years 2010―2015. (A) Incidence of all IPD. (B) Incidence of vaccine-type IPD.

**Fig 3:**
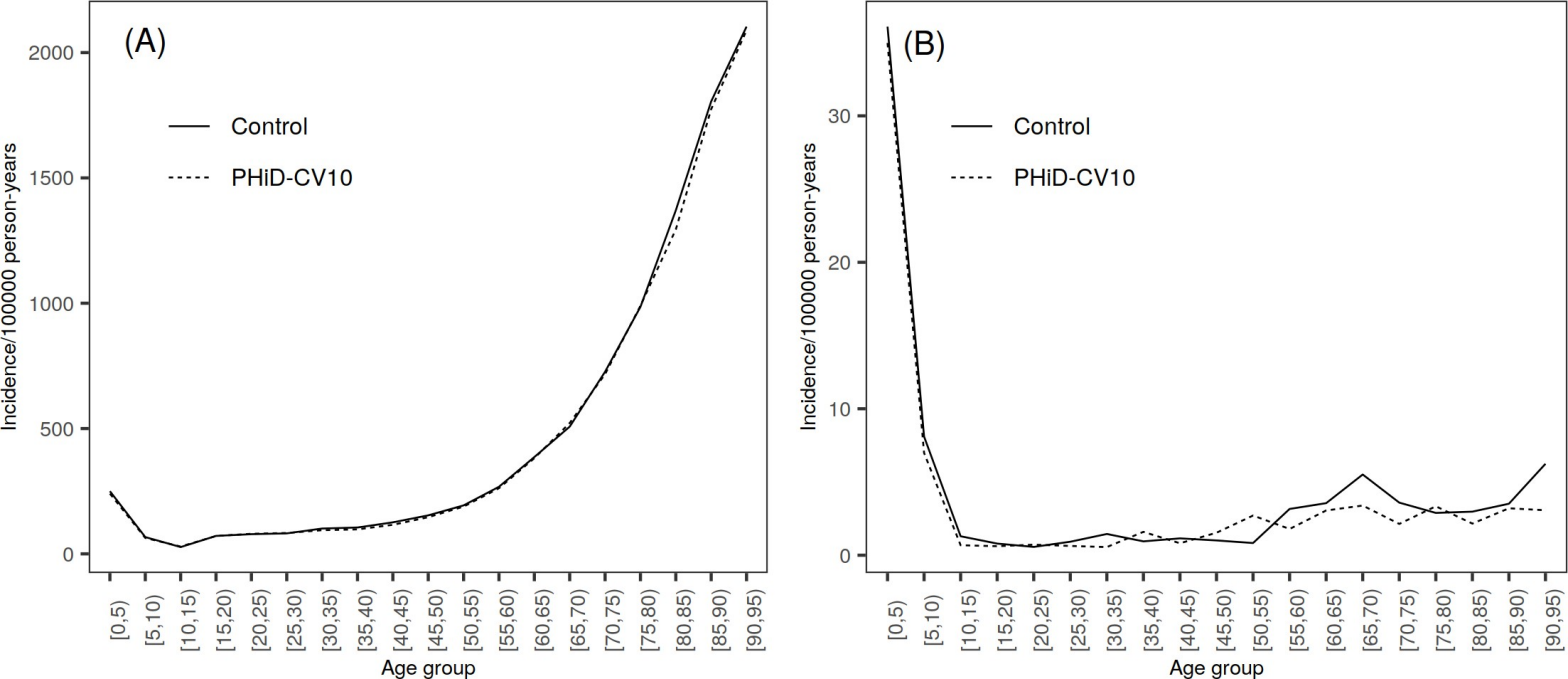
Rates of clinically suspected IPD by age group (in years) in PHiD-CV10 and control clusters, average over years 2010―2015. (A) Incidence of hospital-diagnosed non-laboratory-confirmed IPD or unspecified sepsis. (B) Incidence of hospital-diagnosed non-laboratory-confirmed IPD.

**Fig 4:**
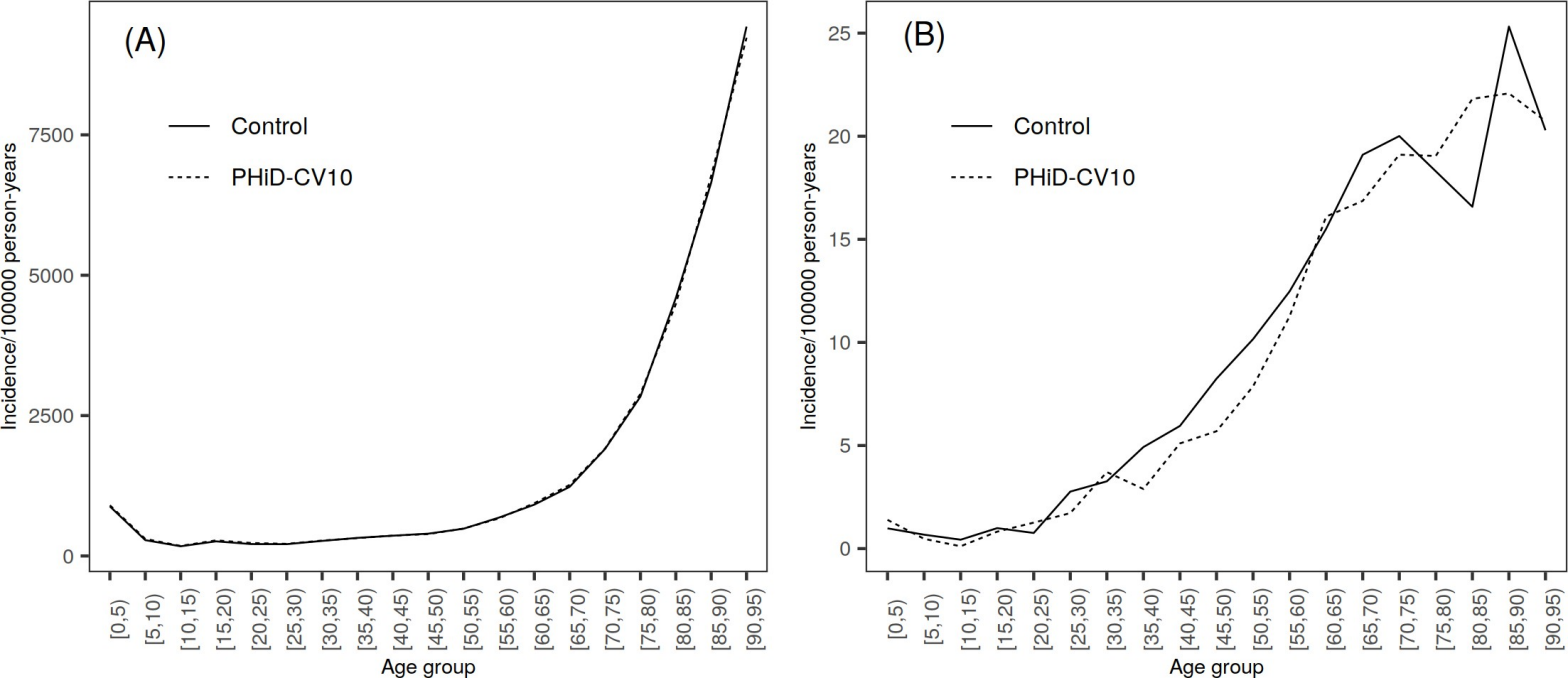
Incidence of pneumonia by age group (in years) in PHiD-CV10 and control clusters, average over years 2010―2015. (A) Incidence of hospital-diagnosed pneumonia. (B) Incidence of hospital-diagnosed empyema.

The unvaccinated population of the study clusters was divided into three age groups (S2 Fig). Population ≥5 years of age (5―7 years for TTPs and outpatient antimicrobial prescriptions) and children <5 years of age were mutually exclusive. Children <3 months of age, a subgroup who were too young to be vaccinated during the PCV-NVP, were evaluated separately. For additional analyses, population was further split into five-year age groups (0―4, 5―9, etc. in Figs 2A, 2B, 3A, 3B, 4A and 4B).

### Follow-up

Per year, follow-up time was calculated as the number of Finnish population in study clusters in each year and age strata. The follow-up time of the children vaccinated within the FinIP trial was removed.

Relocations between clusters or abroad were not estimated during single calendar-years because we assumed they were similar between treatment arms. Follow-up time was aggregated by cluster (1―72), calendar-year (2010―2015) and age-group.

### Ethics statement

The study protocols were approved by the independent ethics committee at the hospital district of Helsinki and Uusimaa and the competent authorities prior to trial start. The study was conducted in accordance with Good Clinical Practice principles and the Declaration of Helsinki. Study protocols are available at https://thl.fi/en/web/thlfi-en/research-and-development/research-and-projects/finip-trial and https://www.gsk-studyregister.com/.

### Statistical analysis

Incidence rates were calculated as arithmetic means for each study year separately, except those of age group <3 months which were calculated for the six follow-up years combined. To increase precision, we combined all ages 5 years and above and analysed PHiD-CV10/control 2+1 and 3+1 clusters combined. For illustration, incidence rates were calculated for 5-year age groups over the whole follow-up period.

The indirect vaccine impact was defined as (1-incidence rate ratio [IRR]) * 100%. To estimate IRRs and consider the possible overdispersion in the cluster-specific incidences, a negative binomial model was used. The cases were grouped by cluster, and the cluster-specific person-years were used as an offset in the model. The factors used in the stratified randomisation (urban vs. rural clusters, cluster size below vs. above average, and TAUVRC trial enrolment in the cluster) were included as explanatory variables. Results are presented as point estimates and 95% confidence intervals (CIs). Relative rate reduction was considered statistically significant if the 95% CI did not include 0%. Bold font indicates statistical significance in tables. No correction for multiple testing was applied.

Wide variations in the incidence rates of several outcomes in the trial clusters were observed. Therefore, we obtained historical aggregate data for the Finnish population in years 2004―2008 to perform explorative post-hoc analyses. We used the incidence of selected outcomes (all IPD, vaccine-type [VT]-IPD, hospital-diagnosed pneumonia [HDP], hospital-treated primary pneumonia [HTPP], TTP, and outpatient antimicrobial purchase) by cluster from these years as an additional explanatory variable to adjust for the background variation between clusters. The statistical program R version 3.4.4 [29] was used in the analyses. The analyses were also performed using Statistical Analysis System (SAS) [30]. The results of PHiD-CV10 impact for years from 2009 till 2012 on probable or culture-confirmed IPD and on HDP in the unvaccinated population, as well as on TTP and on outpatient antimicrobial prescriptions in unvaccinated children ≤7 years of age can be found at ClinicalTrials.gov (NCT00861380, NCT00839254).

## Results

In the 72 clusters considered for the indirect impact analysis, 41188 children were enrolled in the FinIP trial and 4210 in the TAUVRC trial from February 2009 to October 2010 with a total of 45398 children.

The annual number of episodes in 2010―2015 in the unvaccinated population across age groups ranged from 572 to 628 for laboratory-confirmed IPD, from 10100 to 14961 for non-laboratory-confirmed IPD or unspecified sepsis, from 35103 to 42223 for HDP, from 7966 to 9455 for TTPs (children ≤7 years of age), and from 216971 to 255554 for outpatient antimicrobial purchases (children ≤7 years of age). The annual follow-up time ranged from 187065 to 223950 person-years in children <5 years of age, and from 3981437 to 4032499 person-years in age-group ≥5 years. The numbers of episodes in age groups <5, 5―7 and ≥5 years as well as the corresponding person-years are presented in (S1–S3 Tables).

### Vaccination coverage

The trial vaccination coverage increased steadily in time up to 38% in the trial eligible cohort (a 30-month birth cohort from December 2007 through May 2010, S2 and S3 Figs). The final vaccination coverage varied from 29 to 61% in the individual PHiD-CV10 clusters. After the introduction of PHiD-CV10 in the PCV-NVP in September 2010, the vaccination coverage in the population aged <5 years increased to 95% in 2015 (S2 Fig).

### Laboratory-confirmed IPD

The annual incidence of all IPD decreased in PHiD-CV10 and control clusters in children <5 years of age during follow-up (S4 Table) but remained stable in the older population ≥5 years (Table 2). There were no clear differences in all IPD incidence rates between the treatment arms in any of the age groups (Fig 2A, Table 2, S4 and S5 Tables).

**Table 2:** Incidence rates of laboratory-confirmed IPD and the corresponding relative rate reductions in the unvaccinated population ≥5 years of age in PHiD-CV10 vs. control clusters in years 2010 through 2015, Finland.

Outcome definition	Year	Incidence / 100 000 person-years		Relative rate reduction, %	
		PHiD-CV10 clusters	Control clusters	Estimate	95% confidence interval
**All IPD**	2010	14.7	13.6	-8	-28 to 10
	2011	14.5	13.7	-6	-26 to 12
	2012	14	15.1	7	-12 to 22
	2013	13.2	14.2	8	-14 to 25
	2014	12.9	14.4	9	-13 to 27
	2015	15.5	13.8	-11	-33 to 7
**Vaccine-type IPD**	2010	8.5	7.6	-11	-40 to 12
	2011	7.8	7.8	-1	-29 to 21
	2012	**5.9**	**8.6**	**31**	**12 to 46**
	2013	4.2	5.6	24	-4 to 44
	2014	3.4	3.9	11	-27 to 37
	2015	3.4	3.2	-5	-52 to 27
**Vaccine-related type IPD**	2010	1.6	1.8	10	-50 to 44
	2011	2.3	2	-13	-88 to 31
	2012	3	2	-45	-127 to 5
	2013	2.7	2.8	5	-42 to 36
	2014	3.2	2.9	-7	-58 to 26
	2015	5.1	3.8	-34	-88 to 3
**Non-vaccine-related type IPD**	2010	4	3.3	-19	-73 to 18
	2011	4.2	4	-5	-46 to 24
	2012	5	4.3	-16	-62 to 15
	2013	5	4.9	-2	-38 to 24
	2014	5	6.6	21	-12 to 45
	2015	6.9	6.7	-1	-34 to 23

Vaccine-type IPD: serotypes included in PHiD-CV10; in the data 1, 4, 6B, 7F, 9V, 14, 18C, 19F, 23F. Note that there were no cases of serotype 5 in the data.

Vaccine-related type IPD: serotypes that belong to the same serogroups as the vaccine types; in the data: 6A, 6C, 6D, 7B, 7C, 9A, 9N, 18B, 19A, 23A, 23B.

Non-vaccine-related type IPD: in the data 3, 8, 10, 10A, 10F, 11, 11A, 11B, 11C, 12F, 13, 15A, 15B, 15C, 16, 16F, 17, 17F, 20, 21, 22F, 24, 24F, 29, 31, 33, 33A, 33F, 34, 35B, 35F, 38, 40.

The incidence of VT-IPD decreased from 2010 to 2015 in both PHiD-CV10 and control clusters in <5 and ≥5 age groups with a faster decline in PHiD-CV10 clusters (Table 2 and S4 Table). The incidence was lower in PHiD-CV10 clusters compared to control clusters in age group ≥5 years in 2012 (Table 2) mainly due to reductions in serotype 23F, 19F, 4 and 9V IPD. The incidence of VT-IPD was lower in PHiD-CV10 clusters also in years 2013 and 2014 in age group ≥5 years, although CIs included 0%. In children <5 years, a lower incidence of VT-IPD was seen in PHiD-CV10 clusters compared to control clusters in 2013 (S4 Table). Also in infants <3 months of age, the incidence of VT-IPD was lower in PHiD-10 clusters compared to control clusters in years 2010―2015, but the 95% CI included 0% (S5 Table). When aggregating study years, there was a trend for lower VT-IPD incidence rates between the treatment arms in the population older than 70 years of age (Fig 2B).

The incidence rates of vaccine-related and non-vaccine-related IPD increased during follow-up in both PHiD-CV10 and control clusters in age group ≥5 years. There were no clear differences in the incidence rates of these serotype groups between the treatment arms: all 95% CIs included 0% (Table 2). However, in 2014 the incidence of vaccine-related IPD in children aged <5 years was 7.7 per 100000 person-years in the PHiD-CV10 clusters, while there were no cases in the control clusters (S4 Table). In infants <3 months of age the incidence of vaccine-related IPD was higher in PHiD-CV10 clusters compared to control clusters in years 2010―2015, but the CI was wide and included 0% (S5 Table).

### Clinically suspected IPD

No clear differences were observed in the incidence rates of non-laboratory-confirmed IPD or unspecified sepsis between the treatment arms in any of the age groups (Fig 3A, Table 3, S4 and S5 Tables). Instead, there was a notable increasing trend in the incidence in population ≥5 years from 2010 to 2015 in both PHiD-CV10 and control clusters which was not observed in <5 years (Table 3 and S4 Table).

**Table 3:** Incidence rates of clinically suspected IPD without laboratory confirmation and the corresponding relative rate reductions in the unvaccinated population ≥5 years of age in PHiD-CV10 vs. control clusters in years 2010 through 2015, Finland.

Outcome definition	Year	Incidence / 100 000 person-years		Relative rate reduction, %	
		PHiD-CV10 clusters	Control clusters	Estimate	95% confidence interval
**Non-laboratory-confirmed IPD or unspecified sepsis**	2010	233.5	251.1	4	-11 to 17
	2011	262.3	270.9	-2	-18 to 12
	2012	298.8	298.5	-2	-17 to 11
	2013	319	310	-3	-19 to 11
	2014	354.9	355.2	-1	-16 to 13
	2015	358.7	367.5	2	-13 to 14
**Non-laboratory-confirmed IPD**	2010	1.9	2.4	20	-36 to 52
	2011	2.5	3.4	28	-12 to 53
	2012	2.3	1.7	-37	-132 to 17
	2013	2.3	1.9	-28	-131 to 28
	2014	1.6	2.2	29	-20 to 58
	2015	**1.6**	**2.7**	**40**	**1 to 63**

There were no clear differences in the incidence rates of the more specific outcome, non-laboratory-confirmed IPD, between the treatment arms (Fig 3B, Table 3, S4 and S5 Tables). All 95% CIs included 0% except for non-laboratory confirmed IPD in 2015 in age group ≥5 years that was borderline statistically significant. During that year, the incidence was lower in the PHiD-CV10 clusters compared to the control clusters. In children <5 years, there was a clear decreasing trend in both treatment arms (S4 Table).

### Pneumonia

No indirect impact was seen on HDP or HTPP during the follow-up, neither for children <5 years nor the population ≥5 years (Fig 4A, Table 4 and S6 Table). All 95% CIs included 0%. Only in the age group <3 months the rates of HDP and HTPP were lower in the PHiD-CV10 clusters compared to the control clusters, but CIs were wide and included 0% (S5 Table). Interestingly, the incidence of empyema was lower in PHiD-CV10 clusters compared to control clusters in age group ≥5 years in 2011 (Table 4). In younger age groups, no differences in incidence between the treatment arms were seen (Fig 4B and S6 Table).

**Table 4:** Incidence rates of pneumonia and the corresponding relative rate reductions in the unvaccinated population ≥5 years of age in PHiD-CV10 vs. control clusters in years 2010 through 2015, Finland.

Outcome definition	Year	Incidence / 100 000 person-years		Relative rate reduction, %	
		PHiD-CV10 clusters	Control clusters	Estimate	95% confidence interval
**Hospital-diagnosed pneumonia**	2010	836.8	841.1	1	-8 to 9
	2011	954.2	955.9	0	-9 to 9
	2012	919.8	896.8	-3	-12 to 6
	2013	894.9	876.7	-3	-12 to 6
	2014	945.9	939.4	-1	-10 to 7
	2015	1015.7	988.9	-3	-13 to 6
**Hospital-treated primary pneumonia**	2010	487.7	509.4	5	-6 to 14
	2011	563.9	581.9	3	-8 to 13
	2012	531.5	529.9	-1	-13 to 10
	2013	514.4	500.1	-2	-14 to 9
	2014	541	541.3	0	-11 to 10
	2015	566.7	556.3	-2	-13 to 8
**Empyema**	2010	6.9	8	14	-9 to 32
	2011	**6.6**	**8.7**	**24**	**4 to 40**
	2012	7.5	7.4	-2	-36 to 23
	2013	8.6	9	4	-21 to 23
	2014	8.6	8.6	0	-26 to 20
	2015	9.9	10.4	3	-24 to 23

### Tympanostomy tube placements and outpatient antimicrobial purchases

**Fig 5:**
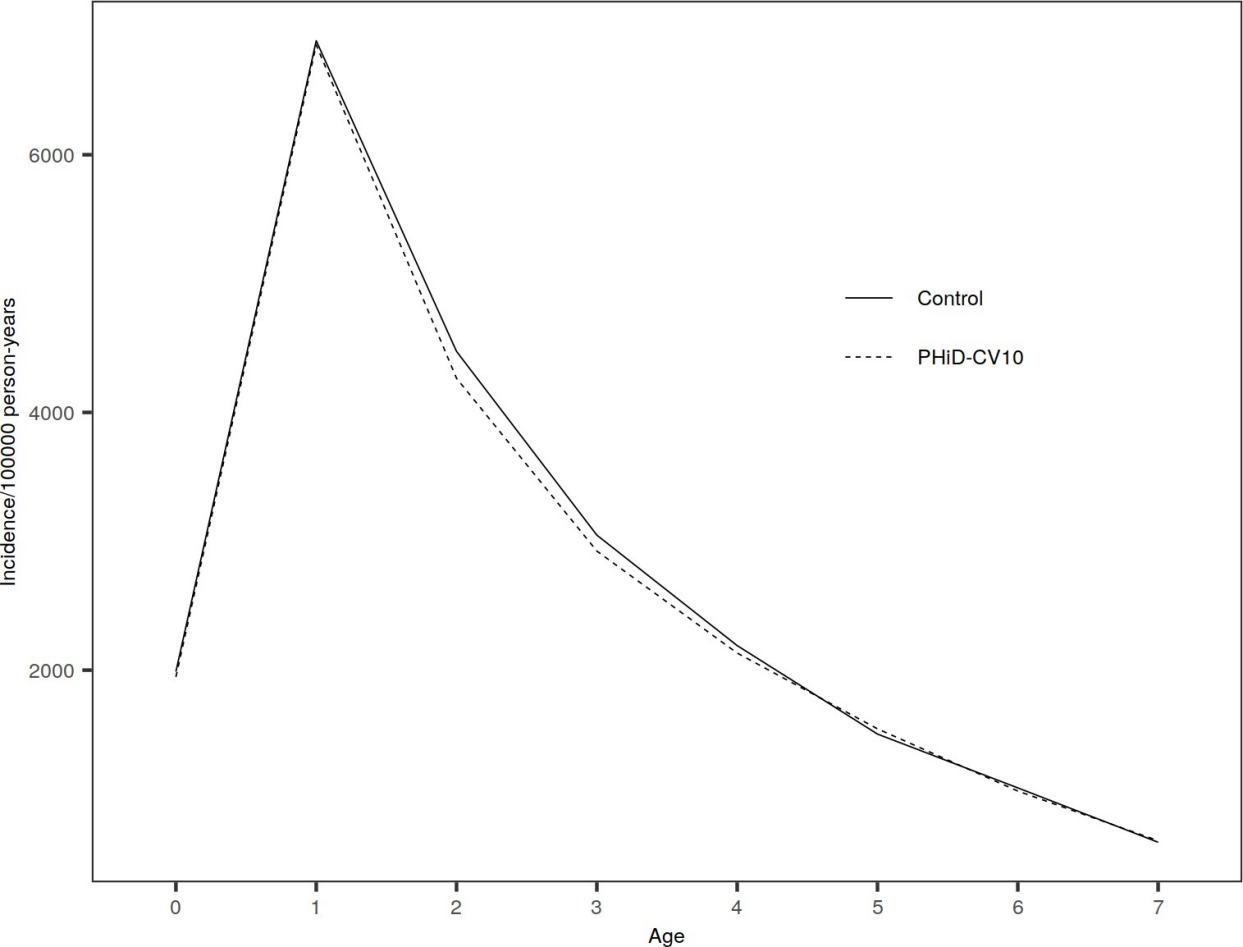
Incidence of tympanostomy tube placements by age year in PHiD-CV10 and control clusters, average over years 2010―2015.

**Fig 6:**
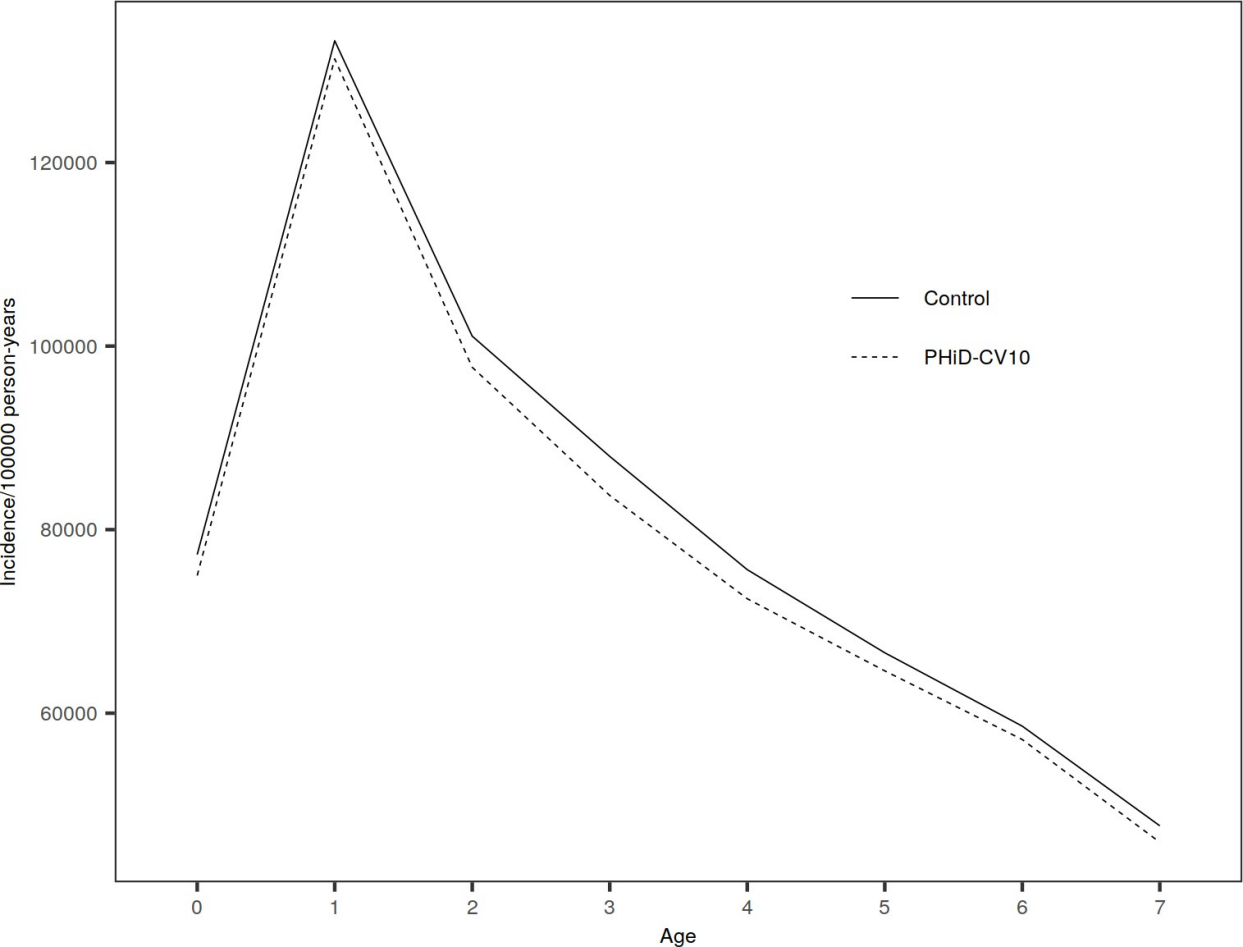
Incidence of outpatient antimicrobial prescriptions by age year in PHiD-CV10 and control clusters, average over years 2010―2015.

Among older children 5―7 years of age, there was an initial increase in the incidence of both TTP and antimicrobial consumption in PHiD-CV10 and control clusters from 2010 to 2011, but a decline after that (Table 5). No indirect impact was seen against neither TTP nor antimicrobial consumption in age groups <5 and 5―7 years (Table 5, S6 Table, Figs 5 and 6). All 95% CIs included 0%. In infants <3 months the rates of TTP and antimicrobial consumption were lower in the PHiD-CV10 clusters compared to the control clusters, but CIs were wide and included 0% (S5 Table).

**Table 5:** Incidence rates of tympanostomy tube placements and outpatient antimicrobial prescriptions and the corresponding relative rate reductions in the unvaccinated population 5―7 years of age in PHiD-CV10 vs. control clusters in years 2010 through 2015, Finland.

Outcome definition	Year	Incidence / 100 000 person-years		Relative rate reduction, %	
		PHiD-CV10 clusters	Control clusters	Estimate	95% confidence interval
**Tympanostomy tube placements**	2010	1099.6	1122.9	0	-18 to 15
	2011	1323.7	1223.6	-10	-31 to 8
	2012	1172.5	1215.3	3	-13 to 16
	2013	900.5	910.5	1	-19 to 17
	2014	878.5	908.5	2	-20 to 20
	2015	1031.7	957.8	-7	-33 to 14
**Antimicrobial prescriptions recommended for acute otitis media**	2010	67219.7	69353.8	2	-4 to 9
	2011	68285.4	69377.8	1	-6 to 7
	2012	59215.6	59247.1	-1	-8 to 6
	2013	48277.5	50791.3	4	-3 to 11
	2014	46672.1	49547	5	-4 to 13
	2015	36628	38245.2	5	-5 to 15

### Additional analyses

We noted substantial variation in the incidence of several outcomes between the study clusters. Therefore, we conducted post-hoc analyses of vaccine impact in population ≥5 years of age to adjust for pre-trial incidence data from years 2004―2008. These cluster-specific historical data highly correlated with the trial data. The post-hoc analyses with pre-trial data did not affect our vaccine impact estimates, although the CIs were narrower in the adjusted analyses (S7 Table). According to this analysis, the reduction in VT-IPD was statistically significant also in 2013, in addition to year 2012.

## Discussion

In our clinical trial setting, we observed reductions in several disease outcomes in the unvaccinated study populations in the PHiD-CV10 clusters compared to the control clusters, although mostly in single follow-up years. The incidence rates of VT-IPD, non-laboratory-confirmed IPD and empyema were lower in PHiD-CV10 clusters compared to control clusters in 2012, 2015 and 2011, respectively, in the age-group ≥5 years. In children <5 years, a lower incidence of VT-IPD was seen in PHiD-CV10 clusters compared to control clusters in 2013. However, consistent indirect impact in the PHiD-CV10 clusters throughout the follow-up years was not observed. It is plausible that the lower than optimal vaccination coverage in the PHiD-CV10 clusters was not high enough for a rapid development of indirect effects. Also the supervening infant PCV-NVP since fall 2010 diluted the vaccine exposure contrasts and reduced the disease burden in both PHiD-CV10 and control clusters within a couple of years.

While we could observe limited indirect impact in the older unvaccinated population, our assessment of the indirect impact on children under 5 years of age was complicated. First, we excluded the children vaccinated within the FinIP trial. This leads to the exclusion of different age strata during consecutive calendar years (S2 Fig). This exclusion does not affect the parallel time comparison of the PHiD-CV10 and control clusters, however it may bias the comparison of incidence rates between calendar years, as the incidence of pneumococcal infection varies by age. Second, children eligible for PCV-NVP were not excluded from the follow-up. The vaccination coverage of PCV-NVP was equally high in the PHiD-CV10 and control clusters and therefore the comparison of the treatment arms should be valid. However, children under 3 months of age were unvaccinated as the first PCV-NVP dose was scheduled at 3 months of age. Interestingly, all point estimates of this age group, except for the vaccine-related and non-vaccine-related type IPD, have positive point estimates, yet the CIs are wide due to the short period of observation.

There is a large number of observational studies showing evidence for indirect impact on unvaccinated populations. Most studies report indirect impact on culture-confirmed IPD after PCV introduction [8–14, 16, 18, 31–39] with varying degrees of replacement disease, but there are also several reports on pneumonia [15, 17, 40–45]. Furthermore, there is an abundance of data showing changes in nasopharyngeal vaccine-type and non-vaccine type carriage prevalence not only in vaccinated, but also in unvaccinated populations [46–51].

Observational studies are, however, prone to many sources of bias: secular trends, changes in diagnostics and treatment, changes in background factors and other interventions relevant to the outcome, patient and physicians’ expectations that change the care-seeking and diagnostics behavior, publication bias, etc. [52]. The corresponding trial design that accounts for most types of bias and allows the estimation of population-level vaccine effects is the cluster-randomised design. Specifically, indirect impact can be estimated by comparing the unvaccinated population in the intervention clusters to the unvaccinated population in the control clusters in parallel follow-up (cf. 19,20). To our knowledge, there are no clinical pneumococcal vaccine trials that have previously reported the indirect impact on disease outcomes.

A specific limitation to assess indirect impact from clinical trials is that the proportion of the target population enrolled and the vaccination coverage achieved are often much lower than in routine vaccination programmes. In our study the vaccination coverage was low during the trial (estimated on average at 38% in the PHiD-CV10 clusters) and the trial enrolment needed to be stopped when the universal immunisations in PCV-NVP started in September 2010. Therefore, the trial vaccination coverage may not have been high enough to induce detectable differences in indirect effects against most of the disease outcomes between the treatment arms. However, our trial setting can be considered representative for the whole population due to the large regional coverage of the clusters and the use of nation-wide health registers.

A small proportion of older adults were concomitantly administered individual pneumococcal vaccinations [24, 25]. However, the uptake of both PCV13 and PPV23 in adults >65 years of age was low during the study follow-up. The randomised study design should take care of any potential confounding related to other pneumococcal vaccinations as the vaccinated individuals were considered randomly distributed between the treatment arms.

Our results should be considered exploratory. We performed statistical testing including several outcomes, multiple calendar years, and several age groups, but did not correct for multiple testing. Only a few findings were considered statistically significant according to the descriptive criterium. However, they all point towards positive indirect impact against vaccine-type disease and none towards negative impact. This suggests true herd effects rather than chance findings that would be expected to be even in either direction, i.e. either positive or negative. On the other hand, replacement by some non-vaccine serotypes was observed. Our results are compatible with those of the two satellite carriage studies conducted in the context of the FinIP trial [53]. In those studies, a reduction of vaccine-type carriage was seen in 2011 in the older unvaccinated siblings of the PHiD-CV10―vaccinated children compared to the siblings of the control children, but the difference was attenuated by 2013.

According to WHO Expert Committee on Biological Standardization, randomisation of groups or clusters, rather than individuals, is preferable when the indirect effects of vaccination are of interest [20]. The FinIP trial was originally designed to assess the total effects in the vaccinated and the indirect effects in the unvaccinated population. Unfortunately, high vaccination coverage was not reached thereby hampering the possibility to develop indirect effects before the supervening PCV-NVP. This also suggests that our previous results from the trial regarding the total effects are predominantly due to the direct effects of the vaccine [21, 23, 26–28]. With regard to the indirect effects, despite many sources of bias, long-term observational studies during PCV-NVP better describe the indirect impact of pneumococcal vaccinations than the results from our trial design. As an example, we observed a marked reduction in HTPP in young unvaccinated children three years after the introduction of PHiD-CV10 in PCV-NVP [41], yet we saw no indirect impact within the trial setting against this outcome. Neither were we able to show consistent indirect impact against most other pneumococcal disease outcomes in the PHiD-CV10 clusters, although some trends towards decreasing incidence were observed e.g. in VT-IPD in 2012―2013. PHiD-CV10 has been shown to reduce vaccine-type carriage and IPD, non-laboratory-confirmed IPD, and pneumonia in unvaccinated populations subject to the infant vaccination programmes [12, 41, 49, 53–55], as well as to decrease the transmission of vaccine-type carriage within families [50].

In conclusion, although no consistent evidence of the indirect impact was seen in this clinical trial setting, several observational studies have shown that PCVs induce herd effects in unvaccinated populations. The development of the indirect effects takes a few years, and high vaccination coverage with catch-up vaccinations speeds up the development. Not all of these prerequisites were fulfilled in this trial because the average vaccination coverage reached only 38% in the PHiD-CV10 clusters. Moreover, PCV-NVP started right after the trial enrolment with high vaccination coverage among infants. It caused strong indirect effects, levelled out the possible differences between the treatment arms, and eliminated the potential to observe indirect impact in the trial setting. Nevertheless, our study contributes to the evidence for the overall impact of PHiD-CV10 against several pneumococcal disease syndromes.

## Supporting information

S1 Checklist CONSORT 2010 checklist of information to include when reporting a randomised trial*. (PDF)

S1 Fig Map of Finland with trial municipalities and treatment arms. Treatment arms are indicated with different colours and the lines represent the boundaries of Finnish municipalities, the number of which ranged from 1 to 12 per cluster. Six biggest cities included several clusters. In the trial, there were altogether 78 clusters which all are presented in the Figure. For the indirect effect analysis, 72 clusters were included.
(TIFF)

S2 Fig Age groups that were evaluated in this study, the FinIP participant cohort, and the infant cohort eligible for PHiD-CV10 in the PCV-NVP. Age groups of the study were children less than 5 years and population 5 years or older (5―7 years for TTPs and outpatient antimicrobial prescriptions). Children under 3 months of age, a subgroup who were too young to be vaccinated during the PCV-NVP, were evaluated separately.
(TIFF)

S3 Fig FinIP trial enrolment by calendar month (February 2009 through October 2010). Solid line: all children; dashed line: infants 6 weeks to 6 months of age; dotted line: catch-up children 7 to 18 months of age.
(TIFF)

S1 Table Number of episodes of laboratory-confirmed and clinically suspected IPD, pneumonia, tympanostomy tube placements and outpatient antimicrobial purchases, and the corresponding person-time in the unvaccinated population <5 years of age in PHiD-CV10 and control clusters in years 2010 through 2015, Finland. (DOCX)

S2 Table Number of episodes of tympanostomy tube placements and outpatient antimicrobial purchases, and the corresponding person-time in the unvaccinated population 5―7 years of age in PHiD-CV10 and control clusters in years 2010 through 2015, Finland. (DOCX)

S3 Table Number of episodes of laboratory-confirmed and clinically suspected IPD and pneumonia, and the corresponding person-time in the unvaccinated population ≥5 years of age in PHiD-CV10 and control clusters in years 2010 through 2015, Finland. (DOCX)

S4 Table Incidence rates of laboratory-confirmed and clinically suspected IPD, and the corresponding relative rate reductions in the unvaccinated population <5 years of age in PHiD-CV10 vs. control clusters in years 2010 through 2015, Finland. Results obtained from explorative post-hoc analyses using data from year 2004―2008 to adjust the impact analyses for the background variation detected in the study clusters.
(DOCX)

S5 Table Incidence rates of all outcomes and the corresponding relative rate reductions in the unvaccinated population <3 months of age in PHiD-CV10 vs. control clusters, average over years 2010―2015, Finland. (DOCX)

S6 Table Incidence rates of pneumonia, tympanostomy tube placements and outpatient antimicrobial purchases, and the corresponding relative rate reductions in the unvaccinated population <5 years of age in PHiD-CV10 vs. control clusters in years 2010 through 2015, Finland. (DOCX)

S7 Table Incidence rates of all IPD, vaccine-type IPD, hospital-diagnosed pneumonia, hospital-treated primary pneumonia, tympanostomy tube placements and outpatient antimicrobial purchases, and the corresponding relative rate reductions in the unvaccinated population ≥5 years of age (5―7 years of age for tympanostomy tube placements and outpatient antimicrobial purchases) in calendar years 2010 through 2015, Finland. Results obtained from explorative post-hoc analyses using data from year 2004―2008 to adjust the impact analyses for the background variation detected in the study clusters.
(DOCX)
