# Supplementary figures and images for: Evaluation of the indirect impact of the 10-valent pneumococcal Haemophilus influenzae protein D conjugate vaccine in a cluster-randomised trial
Source: PLoS One. 2022 Jan 5;17(1):e0261750. doi: 10.1371/journal.pone.0261750 (PMC8730423; doi:10.1371/journal.pone.0261750)

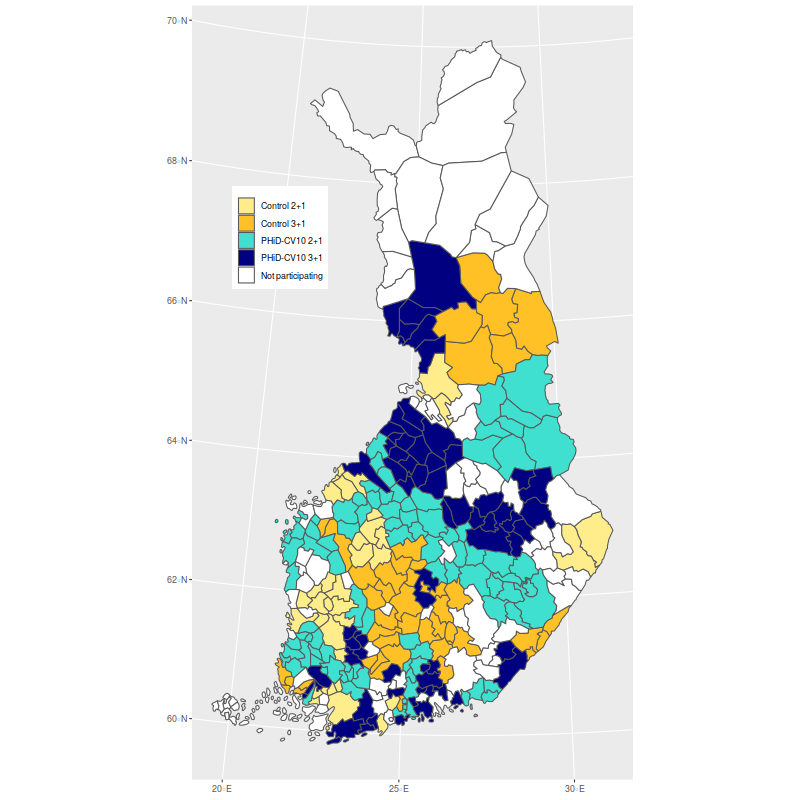

Supplement: S1 Fig — Treatment arms are indicated with different colours and the lines represent the boundaries of Finnish municipalities, the number of which ranged from 1 to 12 per cluster. Six biggest cities included several clusters. In the trial, there were altogether 78 clusters which all are presented in the Figure. For the indirect effect analysis, 72 clusters were included. (TIFF) [file pone.0261750.s002.tiff]

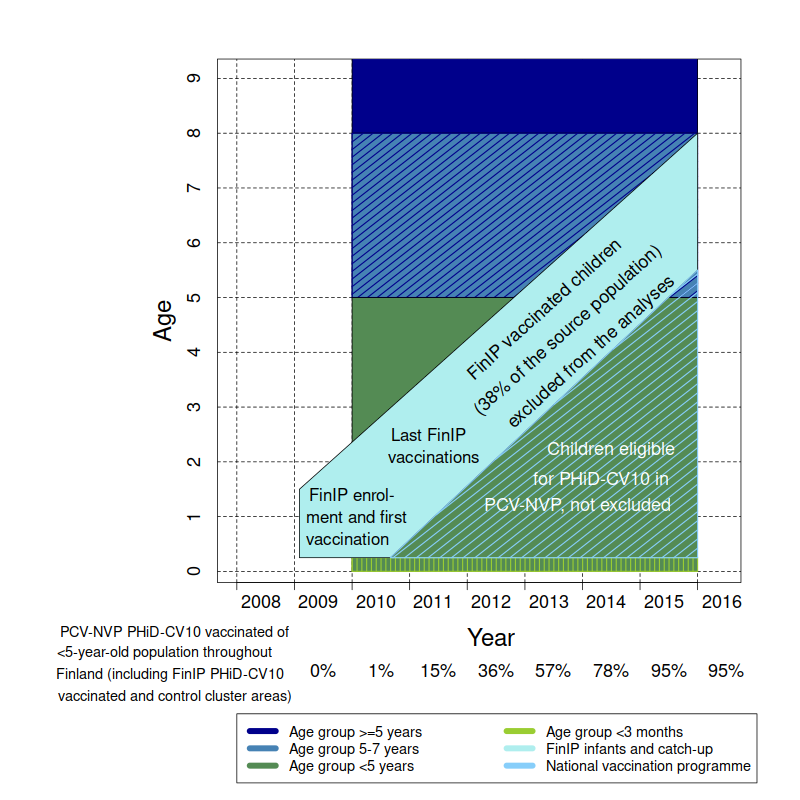

Supplement: S2 Fig — Age groups of the study were children less than 5 years and population 5 years or older (5―7 years for TTPs and outpatient antimicrobial prescriptions). Children under 3 months of age, a subgroup who were too young to be vaccinated during the PCV-NVP, were evaluated separately. (TIFF) [file pone.0261750.s003.tiff]

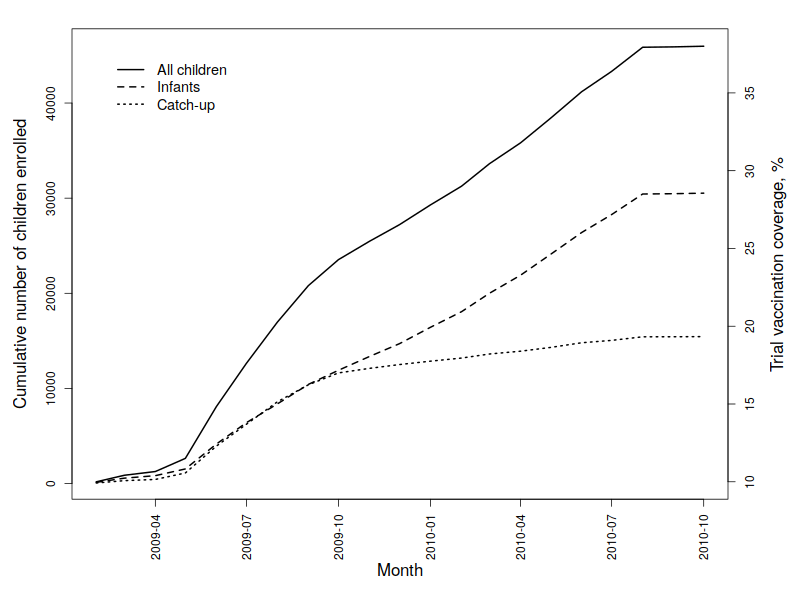

Supplement: S3 Fig — Solid line: all children; dashed line: infants 6 weeks to 6 months of age; dotted line: catch-up children 7 to 18 months of age. (TIFF) [file pone.0261750.s004.tiff]
