# Supplementary material for: Evaluation of the indirect impact of the 10-valent pneumococcal Haemophilus influenzae protein D conjugate vaccine in a cluster-randomised trial
Source: PLoS One. 2022 Jan 5;17(1):e0261750. doi: 10.1371/journal.pone.0261750 (PMC8730423; doi:10.1371/journal.pone.0261750)
Supplement: S1 Table — (DOCX) [file pone.0261750.s005.docx]

| **Outcome** | **PHiD-CV10 clusters** | | | | | | **Control clusters** | | | | | |
| --- | --- | --- | --- | --- | --- | --- | --- | --- | --- | --- | --- | --- |
|  | **2010** | **2011** | **2012** | **2013** | **2014** | **2015** | **2010** | **2011** | **2012** | **2013** | **2014** | **2015** |
| Vaccine-type IPD | 36 | 19 | 9 | 2 | 2 | 1 | 15 | 8 | 6 | 6 | 3 | 2 |
| Vaccine-related type IPD | 2 | 5 | 1 | 6 | 9 | 7 | 1 | 4 | 1 | 1 | 0 | 3 |
| Non-vaccine-related type IPD | 4 | 5 | 3 | 5 | 6 | 1 | 0 | 1 | 0 | 2 | 3 | 3 |
| All IPD | 43 | 29 | 13 | 17 | 17 | 9 | 16 | 13 | 7 | 10 | 6 | 8 |
| Non-laboratory-confirmed IPD or unspecified sepsis | 338 | 403 | 299 | 250 | 284 | 321 | 227 | 219 | 150 | 145 | 137 | 140 |
| Non-laboratory-confirmed IPD | 77 | 89 | 41 | 21 | 21 | 27 | 44 | 45 | 17 | 17 | 14 | 10 |
| Hospital-diagnosed pneumonia | 1151 | 1150 | 1296 | 1095 | 1188 | 1236 | 576 | 601 | 626 | 546 | 614 | 628 |
| Hospital-treated primary pneumonia | 556 | 571 | 604 | 501 | 523 | 519 | 284 | 297 | 288 | 224 | 282 | 277 |
| Empyema | 1 | 1 | 2 | 1 | 4 | 2 | 2 | 2 | 0 | 0 | 0 | 0 |
| Tympanostomy tube placements | 4285 | 4651 | 5079 | 4881 | 4573 | 4967 | 2421 | 2324 | 2751 | 2375 | 2369 | 2803 |
| Antimicrobial prescriptions recommended for acute otitis media | 140284 | 133414 | 132050 | 109868 | 113704 | 95518 | 74740 | 70535 | 69190 | 57767 | 62370 | 51583 |
| Person-time (years) | 126597 | 122990 | 124183 | 127954 | 138969 | 148449 | 65839 | 64075 | 64262 | 66183 | 71435 | 75501 |
