# Supplementary material for: Evaluation of the indirect impact of the 10-valent pneumococcal Haemophilus influenzae protein D conjugate vaccine in a cluster-randomised trial
Source: PLoS One. 2022 Jan 5;17(1):e0261750. doi: 10.1371/journal.pone.0261750 (PMC8730423; doi:10.1371/journal.pone.0261750)
Supplement: S2 Table — (DOCX) [file pone.0261750.s006.docx]

| **Outcome** | **PHiD-CV10 clusters** | | | | | | **Control clusters** | | | | | |
| --- | --- | --- | --- | --- | --- | --- | --- | --- | --- | --- | --- | --- |
|  | **2010** | **2011** | **2012** | **2013** | **2014** | **2015** | **2010** | **2011** | **2012** | **2013** | **2014** | **2015** |
| Tympanostomy tube placements | 963 | 1177 | 1052 | 780 | 665 | 658 | 519 | 575 | 573 | 416 | 359 | 317 |
| Antimicrobial prescriptions recommended for acute otitis media | 58871 | 60716 | 53130 | 41816 | 35328 | 23361 | 32056 | 32602 | 27935 | 23205 | 19578 | 12658 |
| Person-time (years) | 87580 | 88915 | 89723 | 86616 | 75694 | 63779 | 46221 | 46992 | 47150 | 45687 | 39514 | 33097 |
