# Supplementary material for: Evaluation of the indirect impact of the 10-valent pneumococcal Haemophilus influenzae protein D conjugate vaccine in a cluster-randomised trial
Source: PLoS One. 2022 Jan 5;17(1):e0261750. doi: 10.1371/journal.pone.0261750 (PMC8730423; doi:10.1371/journal.pone.0261750)
Supplement: S3 Table — (DOCX) [file pone.0261750.s007.docx]

|  | **PHiD-CV10 clusters** | | | | | | **Control clusters** | | | | | |
| --- | --- | --- | --- | --- | --- | --- | --- | --- | --- | --- | --- | --- |
| **Year** | **2010** | **2011** | **2012** | **2013** | **2014** | **2015** | **2010** | **2011** | **2012** | **2013** | **2014** | **2015** |
| Vaccine-type IPD | 222 | 206 | 157 | 112 | 91 | 89 | 103 | 106 | 117 | 77 | 53 | 43 |
| Vaccine-related type IPD | 43 | 61 | 79 | 71 | 84 | 136 | 25 | 27 | 28 | 38 | 40 | 52 |
| Non-vaccine-related type IPD | 105 | 110 | 133 | 133 | 134 | 182 | 45 | 54 | 59 | 67 | 90 | 91 |
| All IPD | 385 | 382 | 372 | 350 | 342 | 410 | 184 | 187 | 206 | 195 | 197 | 188 |
| Non-laboratory-confirmed IPD or unspecified sepsis | 6133 | 6915 | 7929 | 8489 | 9442 | 9505 | 3402 | 3687 | 4081 | 4252 | 4867 | 4995 |
| Non-laboratory-confirmed IPD | 48 | 64 | 60 | 61 | 41 | 43 | 32 | 46 | 23 | 26 | 30 | 37 |
| Hospital-diagnosed pneumonia | 21981 | 25161 | 24411 | 23812 | 25166 | 26916 | 11395 | 13009 | 12263 | 12024 | 12870 | 13443 |
| Hospital-treated primary pneumonia | 12810 | 14868 | 14106 | 13689 | 14394 | 15017 | 6901 | 7919 | 7246 | 6859 | 7416 | 7562 |
| Empyema | 180 | 175 | 198 | 228 | 228 | 262 | 109 | 119 | 101 | 124 | 118 | 141 |
| Person-time (years) | 2626735 | 2636783 | 2654010 | 2660947 | 2660576 | 2649933 | 1354702 | 1360966 | 1367343 | 1371552 | 1370022 | 1359332 |
