# Supplementary material for: Evaluation of the indirect impact of the 10-valent pneumococcal Haemophilus influenzae protein D conjugate vaccine in a cluster-randomised trial
Source: PLoS One. 2022 Jan 5;17(1):e0261750. doi: 10.1371/journal.pone.0261750 (PMC8730423; doi:10.1371/journal.pone.0261750)
Supplement: S4 Table — Results obtained from explorative post-hoc analyses using data from year 2004―2008 to adjust the impact analyses for the background variation detected in the study clusters. (DOCX) [file pone.0261750.s008.docx]

| **Outcome definition** | **Year** | **Incidence /**  **100 000 person-years** | | **Relative rate reduction, %** | |
| --- | --- | --- | --- | --- | --- |
|  |  | PHiD-CV10 clusters | Control clusters | Estimate | 95% confidence interval |
| **All IPD** | 2010 | 40.7 | 24.3 | -62 | -206 to 0 |
|  | 2011 | 28.3 | 20.3 | -35 | -169 to 28 |
|  | 2012 | 12.5 | 10.9 | -18 | -236 to 55 |
|  | 2013 | 15.9 | 15.1 | -6 | -140 to 51 |
|  | 2014 | 14.6 | 8.4 | -71 | -382 to 31 |
|  | 2015 | 7.2 | 10.6 | 31 | -85 to 73 |
| **Vaccine-type IPD** | 2010 | 34.1 | 22.8 | -48 | -187 to 21 |
|  | 2011 | 18.5 | 12.5 | -37 | -235 to 38 |
|  | 2012 | 8.7 | 9.3 | 12 | -166 to 68 |
|  | 2013 | **1.9** | **9.1** | **79** | **8 to 97** |
|  | 2014 | 1.7 | 4.2 | 56 | -169 to 94 |
|  | 2015 | 0.8 | 2.6 | 68 | -239 to 100 |
| **Vaccine-related type IPD** | 2010 | 1.9 | 1.5 | -25 | -Inf to 88 |
|  | 2011 | 4.9 | 6.2 | 25 | -205 to 80 |
|  | 2012 | 1 | 1.6 | 38 | -Inf to 98 |
|  | 2013 | 5.6 | 1.5 | -257 | -Inf to 39 |
|  | 2014 | **7.7** | **0** | **-Inf** | **-Inf to -148** |
|  | 2015 | 5.6 | 4 | -40 | -555 to 61 |
| **Non-vaccine-related type IPD** | 2010 | 3.8 | 0 | -Inf | -Inf to 65 |
|  | 2011 | 4.9 | 1.6 | -201 | -Inf to 52 |
|  | 2012 | 2.9 | 0 | -Inf | -Inf to 40 |
|  | 2013 | 4.7 | 3 | - | - |
|  | 2014 | 5.2 | 4.2 | -24 | -490 to 67 |
|  | 2015 | 0.8 | 4 | 82 | -55 to 100 |
| **Non-laboratory-confirmed IPD or unspecified sepsis** | 2010 | 267 | 344.8 | 21 | -1 to 37 |
|  | 2011 | 327.7 | 341.8 | 8 | -18 to 28 |
|  | 2012 | 240.8 | 233.4 | -5 | -39 to 20 |
|  | 2013 | 195.4 | 219.1 | 11 | -16 to 31 |
|  | 2014 | 204.4 | 191.8 | -4 | -35 to 20 |
|  | 2015 | 216.2 | 185.4 | -14 | -45 to 10 |
| **Non-laboratory-confirmed IPD** | 2010 | 62 | 66.8 | 6 | -59 to 45 |
|  | 2011 | 73.7 | 70.2 | -2 | -61 to 35 |
|  | 2012 | 33.6 | 26.5 | -20 | -138 to 38 |
|  | 2013 | 16.7 | 25.7 | 34 | -35 to 67 |
|  | 2014 | 15.4 | 19.6 | 23 | -55 to 60 |
|  | 2015 | 18.5 | 13.2 | -37 | -200 to 32 |
