# Supplementary material for: Evaluation of the indirect impact of the 10-valent pneumococcal Haemophilus influenzae protein D conjugate vaccine in a cluster-randomised trial
Source: PLoS One. 2022 Jan 5;17(1):e0261750. doi: 10.1371/journal.pone.0261750 (PMC8730423; doi:10.1371/journal.pone.0261750)
Supplement: S5 Table — (DOCX) [file pone.0261750.s009.docx]

| **Outcome definition** | **Incidence /**  **100 000 person-years** | | **Relative rate reduction, %** | |
| --- | --- | --- | --- | --- |
|  | PHiD-CV10 clusters | Control clusters | Estimate | 95% confidence interval |
| **All IPD** | 63.5 | 69.3 | 9 | -77 to 51 |
| **Vaccine-type IPD** | 35.3 | 46.2 | 24 | -75 to 66 |
| **Vaccine-related type IPD** | 7.1 | 4.6 | -53 | -2988 to 80 |
| **Non-vaccine-related type IPD** | 21.2 | 13.9 | -43 | -435 to 62 |
| **Non-laboratory-confirmed IPD or unspecified sepsis** | 941.1 | 984.3 | 2 | -23 to 22 |
| **Non-laboratory-confirmed IPD** | 7.1 | 9.2 | 24 | -480 to 87 |
| **Hospital-diagnosed pneumonia** | 689.3 | 790.2 | 13 | -17 to 36 |
| **Hospital-treated primary pneumonia** | 395.2 | 489.8 | 21 | -11 to 44 |
| **Tympanostomy tube placements** | 5 | 14 | 66 | -110 to 95 |
| **Antimicrobial prescriptions recommended for acute otitis media** | 10526 | 11396 | 10 | -1 to 19 |
