# Supplementary material for: Evaluation of the indirect impact of the 10-valent pneumococcal Haemophilus influenzae protein D conjugate vaccine in a cluster-randomised trial
Source: PLoS One. 2022 Jan 5;17(1):e0261750. doi: 10.1371/journal.pone.0261750 (PMC8730423; doi:10.1371/journal.pone.0261750)
Supplement: S6 Table — (DOCX) [file pone.0261750.s010.docx]

| **Outcome definition** | **Year** | **Incidence /**  **100 000 person-years** | | **Relative rate reduction, %** | |
| --- | --- | --- | --- | --- | --- |
|  |  | PHiD-CV10 clusters | Control clusters | Estimate | 95% confidence interval |
| **Hospital-diagnosed pneumonia** | 2010 | 909.2 | 874.9 | -3 | -22 to 13 |
|  | 2011 | 935 | 938 | 0 | -16 to 14 |
|  | 2012 | 1043.6 | 974.1 | -5 | -23 to 9 |
|  | 2013 | 855.8 | 825 | -3 | -23 to 15 |
|  | 2014 | 854.9 | 859.5 | 3 | -17 to 19 |
|  | 2015 | 832.6 | 831.8 | 1 | -17 to 16 |
| **Hospital-treated primary pneumonia** | 2010 | 439.2 | 431.4 | 0 | -19 to 16 |
|  | 2011 | 464.3 | 463.5 | 2 | -20 to 19 |
|  | 2012 | 486.4 | 448.2 | -5 | -28 to 14 |
|  | 2013 | 391.5 | 338.5 | -13 | -40 to 9 |
|  | 2014 | 376.3 | 394.8 | 6 | -13 to 22 |
|  | 2015 | 349.6 | 366.9 | 7 | -12 to 22 |
| **Empyema** | 2010 | 0.8 | 3 | 69 | -422 to 99 |
|  | 2011 | 0.8 | 3.1 | - | - |
|  | 2012 | 1.6 | 0 | - | - |
|  | 2013 | 0.8 | 0 | - | - |
|  | 2014 | 2.9 | 0 | - | - |
|  | 2015 | 1.3 | 0 | - | - |
| **Tympanostomy tube placements** | 2010 | 3385 | 3677 | 6 | -12 to 21 |
|  | 2011 | 3782 | 3627 | -5 | -21 to 9 |
|  | 2012 | 4090 | 4281 | 5 | -11 to 19 |
|  | 2013 | 3815 | 3589 | -7 | -26 to 10 |
|  | 2014 | 3291 | 3316 | 0 | -19 to 15 |
|  | 2015 | 3346 | 3713 | 9 | -9 to 24 |
| **Antimicrobial prescriptions recommended for acute otitis media** | 2010 | 110800 | 113500 | 3 | -4 to 9 |
|  | 2011 | 108500 | 110100 | 2 | -5 to 8 |
|  | 2012 | 106300 | 107700 | 1 | -6 to 8 |
|  | 2013 | 85900 | 87300 | 2 | -5 to 9 |
|  | 2014 | 81800 | 87300 | 6 | -2 to 13 |
|  | 2015 | 64300 | 68300 | 7 | -3 to 15 |
