# Supplementary material for: Evaluation of the indirect impact of the 10-valent pneumococcal Haemophilus influenzae protein D conjugate vaccine in a cluster-randomised trial
Source: PLoS One. 2022 Jan 5;17(1):e0261750. doi: 10.1371/journal.pone.0261750 (PMC8730423; doi:10.1371/journal.pone.0261750)
Supplement: S7 Table — Results obtained from explorative post-hoc analyses using data from year 2004―2008 to adjust the impact analyses for the background variation detected in the study clusters. (DOCX) [file pone.0261750.s011.docx]

| **Outcome definition** | **Year** | **Incidence /**  **100 000 person-years** | | **Relative rate reduction, %** | |
| --- | --- | --- | --- | --- | --- |
|  |  | PHiD-CV10 clusters | Control clusters | Estimate | 95% confidence interval |
| **All IPD** | 2010 | 14.7 | 13.6 | -5 | -25 to 12 |
|  | 2011 | 14.5 | 13.7 | -5 | -26 to 3 |
|  | 2012 | 14 | 15.1 | 11 | -6 to 25 |
|  | 2013 | 13.2 | 14.2 | 9 | -12 to 26 |
|  | 2014 | 12.9 | 14.4 | 7 | -16 to 25 |
|  | 2015 | 15.5 | 13.8 | -7 | -28 to 10 |
| **Vaccine-type IPD** | 2010 | 8.5 | 7.6 | -6 | -35 to 16 |
|  | 2011 | 7.8 | 7.8 | -1 | -29 to 21 |
|  | 2012 | **5.9** | **8.6** | **34** | **16 to 49** |
|  | 2013 | **4.2** | **5.6** | **28** | **3 to 46** |
|  | 2014 | 3.4 | 3.9 | 10 | -29 to 37 |
|  | 2015 | 3.4 | 3.2 | -7 | -56 to 26 |
| **Hospital-diagnosed pneumonia** | 2010 | 836.8 | 841.1 | 2 | -7 to 9 |
|  | 2011 | 954.2 | 955.9 | 1 | -7 to 9 |
|  | 2012 | 919.8 | 896.8 | -2 | -10 to 6 |
|  | 2013 | 894.9 | 876.7 | -1 | -9 to 7 |
|  | 2014 | 945.9 | 939.4 | 0 | -8 to 8 |
|  | 2015 | 1015.7 | 988.9 | -2 | -11 to 7 |
| **Hospital-treated primary pneumonia** | 2010 | 487.7 | 509.4 | 6 | -3 to 14 |
|  | 2011 | 563.9 | 581.9 | 5 | -4 to 13 |
|  | 2012 | 531.5 | 529.9 | 1 | -9 to 10 |
|  | 2013 | 514.4 | 500.1 | 0 | -10 to 8 |
|  | 2014 | 541 | 541.3 | 2 | -7 to 10 |
|  | 2015 | 566.7 | 556.3 | -1 | -10 to 8 |
| **Tympanostomy tube placements** | 2010 | 1100 | 1123 | -1 | -19 to 14 |
|  | 2011 | 1324 | 1224 | -10 | -31 to 8 |
|  | 2012 | 1173 | 1215 | 1 | -13 to 14 |
|  | 2013 | 901 | 911 | 0 | -20 to 16 |
|  | 2014 | 879 | 909 | -1 | -23 to 17 |
|  | 2015 | 1032 | 958 | -10 | -35 to 12 |
| **Antimicrobial prescriptions recommended for acute otitis media** | 2010 | 67220 | 69354 | 3 | -4 to 9 |
|  | 2011 | 68285 | 69378 | 1 | -6 to 8 |
|  | 2012 | 59216 | 59247 | -1 | -8 to 6 |
|  | 2013 | 48278 | 50791 | 5 | -3 to 11 |
|  | 2014 | 46672 | 49547 | 5 | -4 to 13 |
|  | 2015 | 36628 | 38245 | 5 | -5 to 15 |
